# Monocytes to lymphocytes multiplying platelets ratio as an early indicator of acute kidney injury in cardiac surgery with cardiopulmonary bypass: a retrospective analysis

**DOI:** 10.1080/0886022X.2024.2364776

**Published:** 2024-06-24

**Authors:** Qian Li, Hong Lv, Yuye Chen, Jingjia Shen, Jia Shi, Fuxia Yan, Sheng Wang, Chenghui Zhou

**Affiliations:** a Department of Anesthesiology, State Key Laboratory of Cardiovascular Disease, Fuwai Hospital, National Center for Cardiovascular Diseases, Chinese Academy of Medical Sciences and Peking Union Medical College, Beijing, China; b Center for Anesthesiology, Beijing Anzhen Hospital, Capital Medical University, Beijing, China

**Keywords:** Monocyte, lymphocyte, cardiac surgery, cardiopulmonary bypass, acute kidney injury

## Abstract

**Objective:**

The monocyte-to-lymphocyte multiplying platelets ratio (MLPR) is a novel systemic inflammatory marker, deriving from the monocyte-to-lymphocyte ratio (MLR). However, the link between MLPR and acute kidney injury following cardiac surgery (CSA-AKI) with cardiopulmonary bypass (CPB) has not been investigated yet. We comprehensively explored the potential linear and nonlinear relationship between MLPR or MLR and CSA-AKI.

**Methods:**

Data of patients who underwent cardiac surgery with CPB between December 2018 and April 2021 were retrospectively collected at Fuwai Hospital, Beijing, China. MLPR was defined as monocyte count (×10^9^/L) × 1000/(lymphocyte count (×10^9^/L) × platelets (×10^9^/L)). MLR was defined as monocyte count (×10^9^/L)/lymphocyte count (×10^9^/L). Logistic regression and restricted cubic spline (RCS) were used for linear and nonlinear analysis. The primary outcome was postoperative AKI within 48 h of after cardiac surgery.

**Results:**

Of the 2420 patients screened, 2387 eligible patients were enrolled in the final analysis; the mean age was 54.7 years, and 1501 [62.9%] were men. The incidence of AKI was 25.8%. Logistic regression showed that MLPR (odds ratio [OR] = 1.31, 95% confidence interval [CI]: 1.16–1.48, *p* < .001) and MLR (OR = 3.06, 95% CI: 1.29–7.29, *p* = .012) were independent risk factors for AKI. Moreover, in the RCS model with adjustment for age (median: 56), female sex, and history of diabetes, a significant statistical difference was detected between preoperative MLPR, MLR, and AKI (*p* for non-linearity <.001). The subgroup analyses revealed similar results.

**Conclusions:**

The study revealed a nonlinear relationship between MLPR and MLR with AKI. MLPR exhibited a J-shaped curve, and MLR showed a favorable S-shaped curve in relation to AKI. Particularly, MLPR emerges as a promising clinical composite index for early CSA-AKI prediction. These findings emphasize the significance of MLPR as a valuable tool in clinical practice for timely identification and management of CSA-AKI.

## 1. Introduction

Cardiac surgery-related acute kidney injury (CSA-AKI), the most prevalent and clinically significant complication in patients undergoing open-heart surgery, affects approximately 4–42% of cases [1]. It is associated with kidney dysfunction, heightened mortality and morbidity rates, and increased medical costs [2,3]. Early detection of AKI is of paramount practical importance, as it has the potential to improve clinical outcomes and alleviate the financial burden. However, serum creatinine (SCr) level and urine output are insensitive and limited in the early diagnosis of kidney function [4]. Several biomarkers have been evaluated *in vitro* and in clinical studies for early detection of AKI. These include neutrophil gelatinase-associated lipocalin, tissue inhibitor of metalloproteinases-2, kidney injury molecule-1, and cystatin C [5–8]. However, limited biomarkers are widely used in clinical practice. Therefore, it is imperative to identify novel AKI biomarkers.

Inflammation plays a crucial role in the onset and progression of AKI, particularly in patients undergoing cardiac surgery with cardiopulmonary bypass (CPB). Individuals with AKI experience alterations in the morphology and function of vascular endothelial cells and tubular epithelium [9,10]. Systemic inflammation indicators such as the systemic inflammation response index, systemic immune-inflammation index, and aggregate index of systemic inflammation have demonstrated an insightful prognostic association with all-cause mortality [11]. Additionally, the monocyte-to-lymphocyte multiplying platelets ratio (MLPR) and monocyte-to-lymphocyte ratio (MLR) have been reported as novel predictors of AKI in studies with small sample sizes and limited surgical types [12–14]. However, the relationship between MLPR and CSA-AKI has not yet been investigated. Hence, we aimed to explore the linear and nonlinear relationships between MLPR, MLR, and CSA-AKI in patients undergoing cardiac surgery with CPB.

## 2. Materials and methods

### 2.1. Study design and population

**Figure 1.**
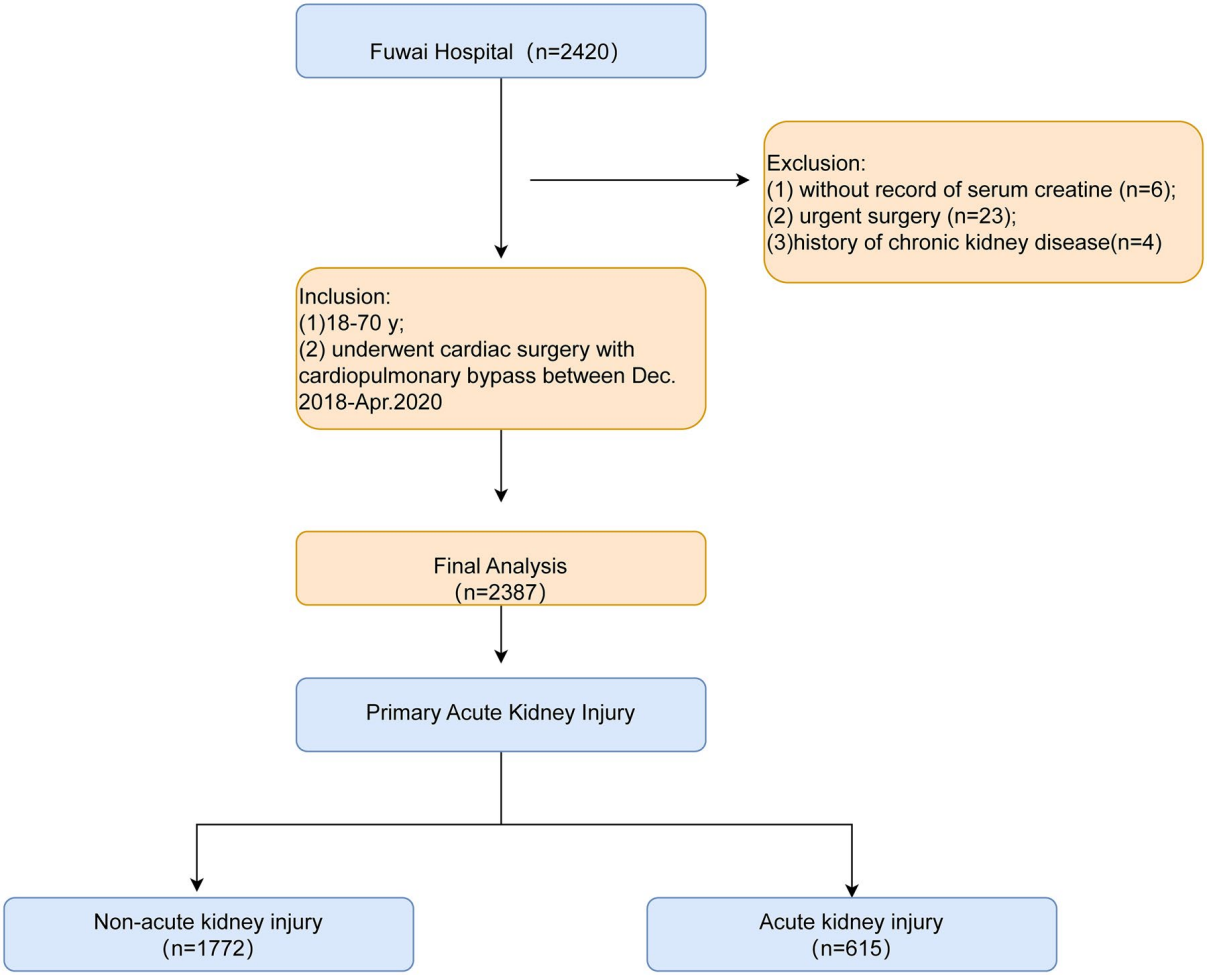
The flow of the present study.

This is a single-center retrospective analysis of 2420 patients aged 18.0–70.0 years who underwent cardiac surgery with CPB between December 2018 and April 2021 in Fuwai Hospital, Beijing, China. Of the 2420 patients, 33 were excluded for the following reasons: (1) lack of serum creatine records, (2) urgent surgery, and (3) a history of chronic kidney disease. Finally, 2387 patients were included in this study. An overview of the design is shown in Figure 1.

### 2.2. Data collection

Patient demographics including age; sex; body mass index (weight (kg)/(height (m^2^)); New York Heart Association (NYHA) classification; medical history such as diabetes, hypertension, hyperlipidemia, previous history of cardiovascular disease; medications including angiotensin-converting enzyme inhibitors, β-blocker, statin; and laboratory test results such as blood routine examination and liver and kidney routine examination. Intraoperative variables were collected from the electronic medical recording system.

### 2.3. Exposure of interest

Exposure of interests were MLPR and MLR, defined using the final preoperative examination values as follows: MLPR = monocyte count (×10^9^/L) × 1000/(lymphocyte count (×10^9^/L) × platelets (×10^9^/L)) [14]. MLR = monocyte count (×10^9^/L)/lymphocyte count (×10^9^/L).

### 2.4. Endpoints

The study endpoint of interest was postoperative AKI, which was ascertained using the Kidney Disease: Improving Global Outcomes criteria based on the perioperative SCr level [15]. AKI was diagnosed when the postoperative SCr level was 1.5-fold higher than the baseline level or when an increase in SCr of 0.3 mg/dL occurred within 48 h postoperatively. We did not use urine output criteria for AKI.

### 2.5. Statistical analysis

Demographic characteristics are presented: as means with standard deviations (SDs) or medians with quartiles for continuous variables and as numbers and percentages for categorical variables. Normality was assessed using the normality test. Continuous variables were analyzed using Student’s *t*-test and Mann–Whitney’s *U*-test, while categorical variables were analyzed using the *χ*^2^ and Fisher’s exact tests.

Logistic regression was employed to evaluate the impact of varying initial-derived inflammatory marker levels on AKI. Additionally, to mitigate the inherent data loss and distortion of dose–response relationships when categorizing continuous variables, we employed restricted cubic splines (RCS) analysis to model the potential nonlinear effects of MLPR, MLR, and CSA-AKI at a constant level. We incorporated RCS with five knots corresponding to the 5th, 35th, 50th, 65th, and 95th percentiles after adjusting for age, sex, and history of diabetes. The median value was used as the reference point (MLPR: 1.0, MLR: 0.2). Moreover, subgroup analyses according to age (>65 years), sex, and history of diabetes were also conducted.

All statistical analyses were performed using R software (version 4.3.1, R Foundation for Statistical Computing, Vienna, Austria) and SPSS software (version 26.0; IBM Corp., Armonk, NY). A two-sided *p* < .05 was considered statistically significant.

## 3. Results

### 3.1. Patient characteristics

Of the 2420 patients screened, 2387 eligible patients were enrolled in the final analysis, among which 62.9% (1501/2387) were men; the mean age was 54.7 years, and the incidence of AKI was 25.8% (615/2387). Patients with higher a MLPR (cutoff >1.0) were older (56.5 [48.5, 63.2] vs. 52.4 [41.2, 60.2], *p* < .001) and exhibited severe heart dysfunction, as indicated by poorer left ventricular end diastolic dimension (*p* < .001). Additionally, the high MLPR group had a higher proportion of individuals with NYHA III–IV, comorbidities of previous coronary heart disease, previous valvular disease, hyperlipidemia, history of infective endocarditis, β-blocker, and stain usage. Laboratory examinations, including neutrophil, alanine aminotransferase, glutamyl transferase, total bilirubin, direct bilirubin, SCr, blood urea nitrogen, prothrombin, D-dimer, high-sensitivity C-reactive protein, and N-terminal brain natriuretic peptide levels, were significantly elevated in the high MLPR group (all *p* < .05). Moreover, there was a notable disparity in the distribution of surgical types between the low and high MLPR groups, with the latter undergoing more valvular surgeries and experiencing longer operative, CPB, and aortic clamp times. Of note, the patients with higher preoperative MLPR were more susceptible to CSA-AKI (385 [32.1%] vs. 230 [19.4%], *p* < .001) and had longer ICU stays (2.0 [1.0–4.0] vs. 2.0 [1.0–3.0], *p* = .007), extended hospital stays (7.0 [6.6–9.0] vs. 7.0 [6.0–8.0], *p* < .001), and a greater likelihood of requiring renal replacement treatment (9 [0.7%] vs. 2 [0.2%], *p* = .036). Details regarding the demographic characteristics of the patients are provided in Table 1, and a breakdown of the demographics based on low and high MLR is presented in Supplementary Table 1.

**Table 1.** Demographics of analysis dataset.

	ALL (*n* = 2387)	MLPR < 1.0 (*n* = 1186)	MLPR ≥ 1.0 (*n* = 1201)	*p* Value
Sex (*n*, %)				<.001
Male	1501 (62.9%)	620 (52.3%)	881 (73.4%)	
Female	886 (37.1%)	566 (47.7%)	320 (26.6%)	
Age (years), median (*Q*1, *Q*3)	54.7 (45.7, 62.1)	52.4 (41.2, 60.2)	56.5 (48.5, 63.2)	<.001
BMI (kg/m^2^), mean ± SD	24.5 ± 3.6	24.2 ± 3.7	24.4 ± 3.4	<.001
NYHA (*n*, %)				<.001
NYHA-I	287(12%)	159 (13.4%)	128 (10.7%)	
NYHA-II	1255(52.6%)	654 (55.1%)	601 (50.0%)	
NYHA-III-IV	845(35.4%)	373(31.5%)	472(39.3%)	
LVEF (*n*, %)				.033
<40%	23 (1.0%)	15 (1.3%)	8 (0.7%)	
40–50%	142 (5.9%)	58 (4.9%)	84 (7.0%)	
>50%	2222 (93.1%)	1113 (93.8%)	1109 (92.3%)	
LVEDD (mm)	51 (45,58)	49 (44,57)	52 (46,60)	<.001
Comorbidities (*n*, %)				
Diabetes	255 (10.7%)	130 (11.0%)	125 (10.4%)	.662
Smoke	822 (34.4%)	352 (29.7%)	470 (39.1%)	<.001
Smoke1m	325 (13.6%)	157 (13.2%)	168 (14.0%)	.593
Myocardial injury	161 (6.7%)	70 (5.9%)	91 (7.6%)	.103
Valvular disease	1697 (71.1%)	812 (68.5%)	885 (73.7%)	.005
Congenital disease	411 (17.2%)	260 (21.9%	151 (12.6%)	<.001
Aortic disease	267 (11.2%)	127 (10.7%	140 (11.7%)	.462
Coronary heart disease	652 (27.3%)	286 (24.1%)	366 (30.5%)	<.001
Peripheral vascular disease	387 (16.2%)	182 (15.3%)	205 (17.1%)	.253
Hyperlipidemia	859 (36.0%)	394 (33.2%	465 (38.7%)	.005
Hypertension	790 (33.1%)	383 (32.3%)	407 (33.9%)	.408
Infective endocarditis	18 (0.8%)	4 (0.3%)	14 (1.2%)	.03
Carotid surgery	14 (0.6%)	6 (0.5%)	8 (0.7%)	.608
Cardiac surgery	142 (5.9%)	67 (5.6%)	75 (6.2%)	.004
Non-invasive tests suggesting carotid artery stenosis >79% or stroke	106 (4.4%)	44 (3.7%)	62 (5.2%)	.085
Mediation (*n*, %)				
Allergy	228 (9.6%)	113 (9.5%)	115 (9.6%)	.968
β-blocker	914 (38.3%)	412 (34.7%)	502 (41.8%)	<.001
ACEI	173 (7.2%)	79 (6.7%)	94 (7.8%)	.272
Statin	206 (8.6%)	75 (6.3%)	131 (10.9%)	<.001
Laboratory results				
Temperature (°C)	36.4 (36.2, 36.5)	36.4 (36.2, 36.5)	36.4 (36.2, 36.5)	.451
HR (bpm)	77 (68, 86)	77 (70, 87)	76 (68, 85)	<.001
PP (mm Hg)	52 (42, 65)	52 (42, 64)	53 (42, 65)	.245
WBC, ×10^9^/L	6.1 (5.2, 7.2)	6.14 (5.23, 7.25)	5.98 (5.07, 7.09)	.018
Neutrophils, %	69.3 (63.3, 74.8)	67.5 (61.5, 73.3)	71 (65.4, 75.9)	<.001
Hemoglobin, dL	138 (127, 149)	138 (127, 149)	138 (128, 148)	.869
Platelets, ×10^9^/L	200 (167, 240)	228 (196, 264)	176 (148, 206)	<.001
Lymphocyte, ×10^9^/L	1.88 (1.52, 2.29)	2.16 (1.8, 2.57)	1.64 (1.34, 1, 96)	<.001
Monocyte, ×10^9^/L	0.39 (0.31, 0.47)	0.35 (0.29, 0.43)	0.43 (0.35, 0.51)	<.001
Baseline creatinine (mg/dL)	82 (72, 93)	80.3 (70, 92)	83.5 (74, 95)	<.001
BUN (mg/dL)	6.0 (4.9, 7.3)	5.7 (4.7, 6.9)	6.2 (5.1, 7.7)	<.001
AST (U/L)	25 (21, 31)	25 (21, 31)	26 (21, 33)	.096
ALT (U/L)	19 (13, 29)	18 (12, 29)	20 (13, 30)	.006
ALP (U/L)	65 (54, 79)	65 (54, 78)	65 (54, 79)	.096
GGT (U/L)	25 (18, 40)	24 (17, 38)	26 (19, 42)	<.001
Total bilirubin (μmol/L)	11.9 (8.8 (16.2)	11.5 (8.6, 15.3)	12.4 (9.3, 17.5)	<.001
Direct bilirubin (μmol/L)	3.3 (2.4, 4.9)	3.0 (2.2, 4.3)	3.6 (2.6, 5.7)	<.001
ALB (mg/dL)	39.8 (37.7, 41.9)	40 (37.9, 42.1)	39.6 (37.5, 41.7)	.001
TP (mg/dL)	67.7 (64.4, 71.6)	68.6 (65, 72.4)	67.1 (63.7, 70.6)	<.001
PT, s	13.1 (12.7, 13.7)	13.1 (12.7, 13.6)	13.2 (12.8, 13.8)	<.001
D-dimer, mg/L	0.24 (0.17, 0.37)	0.23 (0.17, 0.34)	0.25 (0.18, 0.39)	<.001
NT-proBNP, pg/mL	275.2 (82, 783)	215.6 (64.7, 706.6)	387.4 (113.4, 1032)	<.001
hs-CRP, mg/L	0.84 (0.35, 2.18)	0.79 (0.31, 1.93)	0.89 (0.39, 2.47)	<.001
MLR	0.20 (0.16, 0.26)	0.16 (0.14, 0.19)	0.26 (0.21, 0.31)	<.001
MLPR	1.0 (0.74, 1.40)	0.75 (0.61, 0.87)	1.39 (1.16, 1.81)	<.001
Surgery type, *n* (%)				
Valvular	1500 (62.8%)	732 (61.7%)	768 (63.9%)	.26
CABG	706 (29.6%)	305 (25.7%)	401 (33.4%)	<.001
Congenital	341 (14.3%)	223 (18.8%)	118 (9.8%)	<.001
Aortic	185 (7.8%)	84 (7.1%	101 (8.4%)	.225
Perioperative variables				
Surgery time (min)	240 (198, 288)	230.5 (191.8, 275)	250 (203, 297.5)	<.001
CPB time (min)	115 (85, 147)	109 (82, 143)	119 (89, 151)	<.001
Aorta clamp time (min)	81 (58, 109)	76.5 (53, 104)	84 (62, 113)	<.001
Rectal temp	32 (31, 32.9)	32 (31.2, 32.9)	32 (31, 32.9)	.163
Postoperative variables				
Platelets, ×10^9^/L	210 (161, 265)	115 (181, 279)	194 (148, 247)	<.001
Lymphocyte, ×10^9^/L	0.98 (0.70, 1.28)	1.08 (0.82, 1.41)	0.87 (0.62, 1.13)	<.001
Monocyte, ×10^9^/L	0.15 (0.08, 0.26)	0.14 (0.08, 0.25)	0.16 (0.09, 0.28)	.005
MLR	0.15 (0.09, 0.25)	0.13 (0.07, 0.22)	0.18 (0.10, 0.30)	<.001
MLPR	0.73 (0.40, 1.29)	0.57 (0.31, 1.00)	0.92 (0.53, 1.59)	<.001
Urine volume (48 h)	2380 (1905, 2880)	2370 (1880, 2860)	2380 (1950, 2880)	.311
End point				
AKI	615 (25.8%)	230 (19.4%)	385 (32.1%)	<.001
Length of ICU stays	2.0 (1.0–4.0)	2.0 (1.0–3.0)	2.0 (1.0–4.0)	.007
Length of hospital stays	7 (6.0–8.0)	7.0 (6.0–8.0)	7.0 (6.6–9.0)	<.001
Death	3 (0.1%)	–	3 (0.2%)	.25
Renal replace treatment	11 (0.5%)	2 (0.2%)	9 (0.7%)	.036

BMI: body mass index; NYHA: New York Heart Association; LVEF: left ventricular ejection function; LEVDD: left ventricular end-diastolic dimension; ACEI: angiotensin-converting enzyme inhibitors; PP: pulse pressure; WBC: white blood cell; AST: aspartate aminotransferase; ALT: alanine aminotransferase; ALP: alkaline phosphatase; GGT: glutamyl transferase; BUN: blood urea nitrogen; PT: prothrombin time; ALB: albumin; NT-proBNP: N-terminal brain natriuretic peptide; hs-CRP: high-sensitivity C-reactive protein; CABG: coronary artery bypass grafting; CPB: cardiopulmonary bypass.

### 3.2. Linear and nonlinear relationship between MLPR, MLR, and CSA-AKI

A statistically significant difference was detected in the logistic regression model after adjusting for age, sex, and history of diabetes with respect to MLPR and MLR in relation to AKI. We categorized MLPR (MLPR: *Q*1 = 0.75, *Q*2 = 1.0, *Q*3 = 1.4) and MLR (MLR: *Q*1 = 0.16, *Q*2 = 0.2, *Q*3 = 0.26) with the quartile. Compared to an MLPR below 0.75, the odds ratio (OR) was 1.37 (95% confidence interval [CI] 1.02–1.84, *p* = .036) for an MLPR between 0.75 and 1.0; the OR was 1.75 (95% CI 1.31–2.34, *p* < .001) for an MLPR between 1.0 and 1.4; the OR was 3.04 (95% CI 2.28–4.06, *p* < .001) for an MLPR above 1.4. Additionally, compared to an MLR below 0.16, the OR was 1.86 (95% CI: 1.42–2.43, *p* < .001) for and MLR above 0.26.

Furthermore, a notable nonlinear relationship was identified in the RCS model, adjusted for age, female sex, and history of diabetes. A distinction was observed among the preoperative MLPR, MLR, and CSA-AKI (*p* for non-linearity <.001).

**Figure 2.**
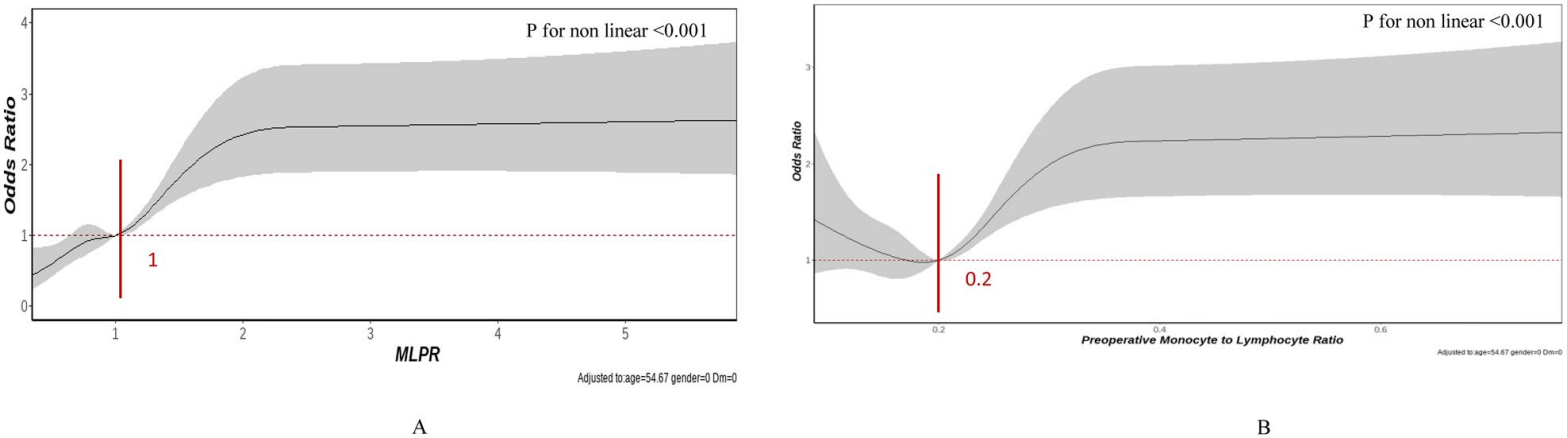
Multivariable-adjusted odds ratios for CSA-AKI, (A) preoperative monocyte × 1000/(lymphocyte × platelets) (MLPR) and (B) preoperative monocyte to lymphocyte ratio (MLR). Grey lines represent references for odds ratios, and grey areas represent 95% confidence intervals. The model was adjusted for age, sex, and previous history of diabetes. The reference point is the median value for MLPR (1.0) and MLR (0.2).

As shown in Figure 2, the MLPR exhibited a somewhat J-shaped correlation with the risk of AKI. Following an MLPR of 1.0, MLPR displayed a moderately favorable relationship with AKI risk, yielding an OR of 1.94 (95% CI 1.59–2.36, *p* < .001) per SD, which subsequently plateaued at a relatively stable level after reaching 2.0 for AKI. Notably, regarding the S-shaped relationship between preoperative MLR and AKI, the risk was relatively steady as the MLR increased, reaching its lowest point around 0.2, followed by a substantial increase (*p* < .001). Compared to an MLR below 0.2, the OR was 1.44 (95% CI: 1.18–1.74, *p* < .001) per SD.

### 3.3. Subgroup analysis of MLPR, MLR, and CSA-AKI

**Figure 3.**
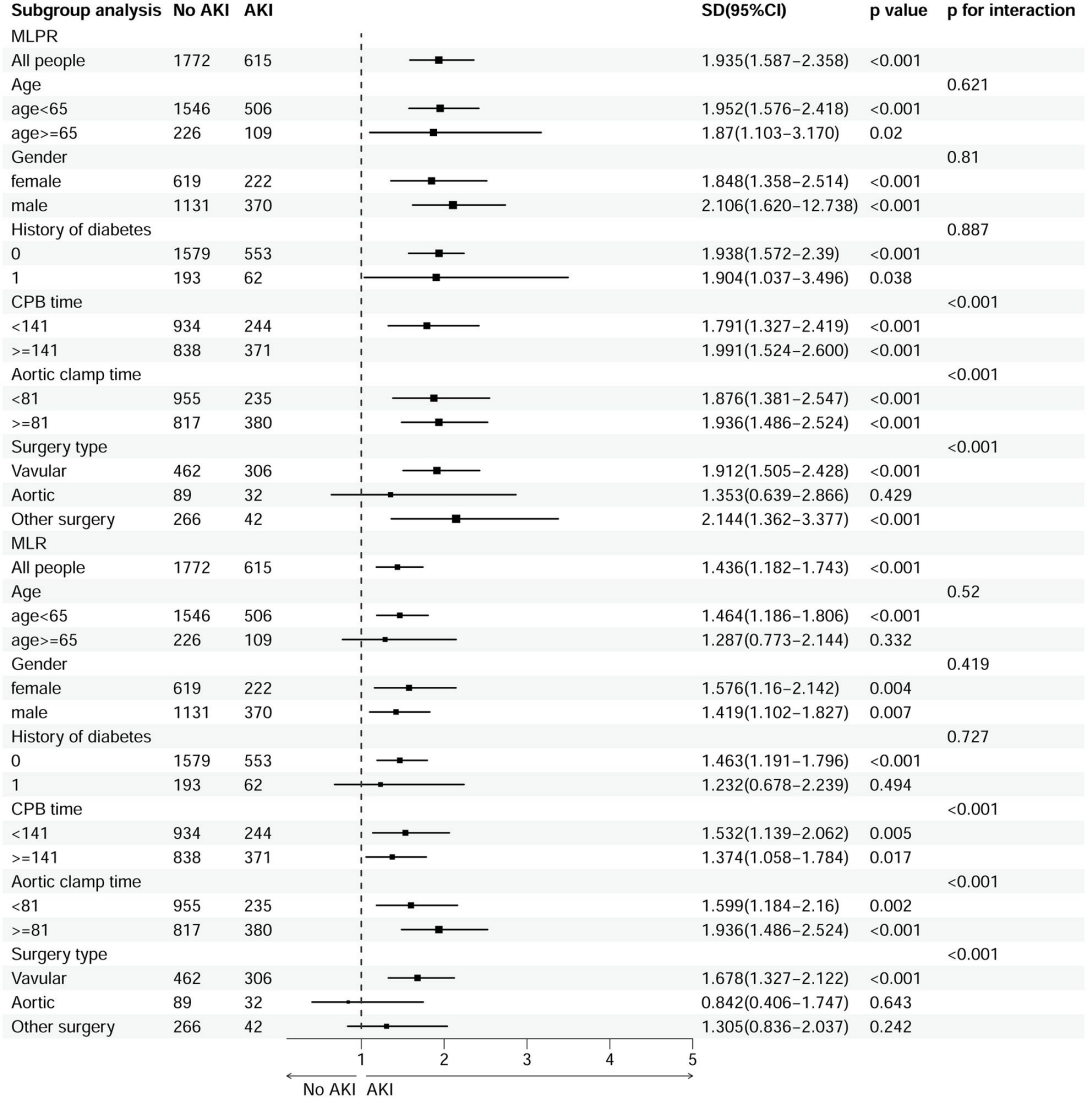
Subgroup analysis for multivariable-adjusted odds ratios of MLPR and MLR for AKI based on logistic regression.

Subgroup analysis, including age (age < 65 years vs. age ≥ 65 years), sex (male vs. female), and history of diabetes mellitus, were conducted. The association of MLR and MLPR with AKI was similar across age, sex, and the presence or absence of diabetic (Figure 3, Supplementary Figures 1 and 2). However, the associations of MLR and MLPR with AKI were stronger among those with longer CPB time, aortic clamp, and valvular surgery (Figure 3).

### 3.4. Relationship between postoperative MLPR, MLR, and CSA-AKI

A logistic regression model, adjusted for age, sex, and diabetes suggested that postoperative MLPR and MLR negatively correlated with CSA-AKI (MLPR: OR = 1.055, 95% CI: 1.002–1.112, *p* = .043; MLR: OR = 1.505, 95% CI: 1.060–2.137, *p* = .022, ORs were per 1 unit of change in MLR and MLPR).

## 4. Discussion

The principal finding of this study was that MLPR and MLR exhibited nonlinear relationships with CSA-AKI, as determined by RCS models (adjusted for age, sex, and history of diabetes). It is noteworthy that MLPR showed a somewhat J-shaped favorable association with the risk of AKI, whereas an S-shaped dose-dependent relationship was observed between MLR and CSA-AKI. The association of MLR and MLPR with AKI were similar across age, sex, and the presence or absence of diabetics. Additionally, the associations of MLR and MLPR with AKI were stronger among those with longer CPB time, aortic clamp, and valvular surgery.

Peripheral blood counts have garnered attention owing to the close association between immune-inflammatory status and the occurrence and development of AKI. Preoperative MLPR has good predictive efficiency for pulmonary infection-related AKI and could be used as a simple clinical composite index [14]. However, no study has explored the relationship between MLPR and CSA-AKI. In the present study, linear and nonlinear relationships between MLPR and AKI were observed. Following an MLPR of 1.0, there was a moderately favorable link between MLPR and the risk of AKI, which then plateaued at a relatively stable level after reaching 2.0 for AKI. In contrast, studies enrolling 331 patients who underwent cardiac valve surgery and 255 patients with aortic surgery showed that an elevated preoperative MLR significantly correlated with an increased risk of postoperative AKI [12,16]. Consistent with previous studies, our study included 2387 patients who underwent cardiac surgery with CPB, further confirming an S-shaped nonlinear relationship between MLR and CSA-AKI. Notably, MLPR outperformed MLR in predicting CSA-AKI, suggesting that MLPR could serve as a novel predictor of CSA-AKI and be incorporated into clinical practice, thus enhancing the early prediction of AKI.

The etiology of CSA-AKI is multifactorial and complex [9]. Accumulating evidence underscores the pivotal role of systemic inflammatory markers in the development of AKI [17]. These inflammatory mediators include blood cells, endothelial cells, macrophages, lymphocytes, and platelets. Inflammation is a complex biological response essential for eliminating microbial pathogens and renovating tissues following diverse forms of injury [10]. Both infectious and noninfectious factors could elevate lymphocyte apoptosis and monocyte proliferation, thereby exerting immune response [18,19]. Furthermore, ischemia–reperfusion injuries caused by CPB, anesthesia, surgical stress, and low temperatures can trigger inflammation and impair kidney function [20]. The nonlinear relationship observed in MLPR, MLR, and CSA-AKI implies that dynamic inflammation pathways are activated in response to alleviate the impact of ischemia–reperfusion injury caused by CPB. Additionally, there were significant interactions between MLPR and MLR with CPB time or aortic clamp time. The associations of MLR and MLPR with AKI were stronger among those with longer CPB time and aortic clamp time.

MLPR can be explained by MLR and platelet count. Monocyte macrophages are important innate immune cells that regulate the immune response during AKI [19]. Peripheral blood monocytes migrate to the injured kidney and transform into macrophages, which can diversify into M1 macrophages with pro-inflammatory effects and M2 macrophages with anti-inflammatory effects. Recent research has aimed to inhibit M1 and activate M2 to reduce damage, enhance repair, suppress inflammation, stimulate collagen remodeling, and prevent advanced kidney fibrosis [21,22]. Preoperative higher monocyte and decreased platelet counts were positively associated with a greater incidence of AKI [23–25]. The connection between platelet count and postoperative AKI may be attributed to impaired kidney microvascular circulation resulting from reduced blood flow owing to microthrombus formation [26]. Additionally, the intricate interplay between immune mechanisms, inflammatory cascade activation, and coagulation pathway disruption contributes to microvascular dysfunction, leukocyte/platelet activation, and microthrombi formation, ultimately inducing kidney tubular epithelial cell injury [27,28]. Additionally, we found that postoperative MLPR and MLR were independent risk factors for CSA-AKI. This may be attributed to that CPB’s association with decreased platelet counts and the activation of both macrophages and platelets [29], as well as surgical stress, which can suppress cellular immunity, resulting in lymphopenia [30]. Cardiovascular surgery with CPB triggers significant systemic inflammatory response and causes platelet injury. Hence, MLPR and MLR, being accessible, can serve as alternative predictors of AKI. The predictive effectiveness of MLPR can be used to comprehensively elucidate the relationship between systemic inflammation, immunity, and coagulation disorders. Therefore, elevated MLPR and MLR could act as early warning indicators of AKI, enabling healthcare providers to closely monitor patients and proactively implement preventive measures.

This study had several limitations. First, this was a single-center retrospective study, and the potential influence of confounding biases could not be eliminated. Second, the diagnosis of AKI relied solely on changes in SCr levels without considering urine volume. Third, data on cytokine examination, such as IL-6, IL-8, IL-10, and tumor necrosis factor-α, were not collected. Fourth, the dynamic changes in MLR and MLPR were not compared owing to data limitations, which could have provided further insight into their relationship with long-term kidney recovery. Fifth, our data primarily consisted of male patients who underwent valvular cardiac surgery, which potentially limits the generalizability of our findings. To mitigate this bias, we conducted a subgroup analysis. Multicenter prospective studies are essential to validate the predictive significance of composite inflammatory indicators for CSA-AKI.

## 5. Conclusions

The study revealed a nonlinear relationship between MLPR and MLR with AKI. MLPR exhibited a J-shaped curve, and MLR showed a favorable S-shaped curve in relation to AKI. Particularly, MLPR emerges as a promising clinical composite index for early CSA-AKI prediction. These findings emphasize the significance of MLPR as a valuable tool in clinical practice for timely identification and management of CSA-AKI.

## Supplementary Material

Supplemental Material

## Data Availability

The original contributions presented in the study are included in the article/Supplementary Material. Further inquiries can be directed to the corresponding author.
