# Supplementary material for: Monocytes to lymphocytes multiplying platelets ratio as an early indicator of acute kidney injury in cardiac surgery with cardiopulmonary bypass: a retrospective analysis
Source: Ren Fail. 2024 Jun 24;46(2):2364776. doi: 10.1080/0886022X.2024.2364776 (PMC11198127; doi:10.1080/0886022X.2024.2364776)
Supplement: Supplemental Material [file IRNF_A_2364776_SM8016.pdf]

Supplementary

Table 1: Demographics of analysis dataset

	ALL(n=2387)	MLR<0.2 (n=1158)	MLPR>=0.2(n=1229)	P value
Sex (n, %)				<0.001
Male	1501(62.9%)	606 (52.3%)	895 (72.8%)	
Female	886(37.1%)	552 (47.7%)	334 (27.2%)	
Age (y), median (Q1, Q3)	54.7(45.7,62.1)	53(41.2,60.4)	55.9(48.4,63.2)	<0.001
BMI (kg/m2), mean ± SD	24.5±3.6	24.1±3.6	24.6±3.5	<0.001
NYHA (n, %)				<0.001
NYHA-I	287(12%)	147 (12.7%)	140 (11.4%)	
NYHA-II	1255(52.6%)	647 (55.9%)	608 (49.5%)	
NYHA-III-IV	845(35.4%)	364(31.4%)	481(39.1%)	
LVEF (n, %)				0.089
<40%	23 (1.0%)	15 (1.3%)	8 (0.7%)	
40-50%	142 (5.9%)	60 (5.2%)	82 (6.7%)	
>50%	2222 (93.1%)	1083 (93.5%)	1139 (92.7%)	
LVEDD(mm)	51(45,58)	50(44,57)	52(46,58)	<0.001
Comorbidities (n, %)				
Diabetes	255 (10.7%)	118 (10.2%)	137 (11.1%)	0.449
Smoke	822 (34.4%)	350 (30.2%)	472 (38.4%)	<0.001
Smoke1m	325 (13.6%)	153 (13.2%)	172 (14.0%)	0.577
Myocardial injury	161 (6.7%)	57 (4.9%)	104 (8.5%)	<0.001
Valvular disease	1697 (71.1%)	846 (73.1%	851 (69.2%)	0.04
Congenital disease	411 (17.2%)	264 (22.8%)	147 (12.0%)	<0.001
Aortic disease	267 (11.2%)	122 (10.5%	145 (11.8%)	0.328
Coronary heart disease	652 (27.3%)	236 (20.4%)	416 (33.8%)	<0.001
Peripherial vascular disease	387 (16.2%)	187 (16.1%)	200 (16.3%)	0.934
Hyperlipidaemia	859 (36.0%)	355 (30.7%)	504 (41.0%)	<0.001
Hypertension	790 (33.1%)	351 (30.3%)	439 (35.7%)	0.005
Infective endocarditis	18 (0.8%)	3 (0.3%)	15 (1.2%)	0.008
Carotid surgery	14 (0.6%)	6 (0.5%)	8 (0.7%)	0.671
Cardiac surgery	142 (5.9%)	62 (5.4%)	80 (6.5%)	0.233
Non-invasive tests suggesting carotid artery stenosis >79% or Stroke	106 (4.4%)	33 (2.8%)	73 (5.9%)	<0.001
Mediation (n, %)				
Allergy	228 (9.6%)	104 (9.0%)	124 (10.1%)	0.357
β-blocker	914 (38.3%)	383 (33.1%)	531 (43.2%)	<0.001
ACEI	173 (7.2%)	73 (6.3%)	100 (8.1%)	0.084
Statin	206 (8.6%)	60 (5.2%)	146 (11.9%)	<0.001
Laboratory results				
Temperature(℃)	36.4(36.2,36.5)	36.4(36.2,36.5)	36.4(36.2,36.5)	0.906
HR(bpm)	77(68,86)	77(69,86)	76(68,86)	0.562
PP (mm Hg)	52(42,65)	52(42,63)	53(42,66)	0.181
WBC, 10^9/L	6.1(5.2,7.2)	5.87(5.0,6.97)	5.26(5.32,7.41)	<0.001
Neutrophils,%	69.3(63.3,74.8)	67.5(61.2,73.2)	71(65.4,75.8)	<0.001

Haemoglobin/DL	138(127,149)	138(126,149)	138(128,148)	0.708
Platelets, 10^9/L	200(167,240)	204(174,244)	197(162,237)	<0.001
Lymphocyte,10^9/L	1.88(1.52,2.29)	2.16(1.59,2.57)	1.65(1.36,2.0)	<0.001
Monocyte,10^9/L	0.39(0.31,0.47)	0.33(0.28,0.40)	0.44(0.37,0.54)	<0.001
Baseline creatinine, (mg/DL)	82(72,93)	80(70,91.7)	83.5(74,95)	<0.001
BUN (mg/DL)	6.0(4.9,7.3)	5.8(4.8,7.2)	6.1(5.0,7.5)	<0.001
AST (U/L)	25(21,31)	25(21,30)	26(21,32)	0.005
ALT (U/L)	19(13,29)	18(12,28)	20(13,31)	<0.001
ALP (U/L)	65(54,79)	63(53,76)	67(55,80)	<0.001
GGT (U/L)	25(18,40)	23(16,37)	28(19,42.5)	<0.001
Total bilirubin, (μmol/L)	11.9(8.8(16.2)	12.0(8.8,16.1)	11.9(8.9,16.2)	0.572
Direct bilirubin ,(μmol/L)	3.3(2.4,4.9)	3.1(2.3,4.6)	3.5(2.5,5.2)	<0.001
ALB (mg/DL)	39.8(37.7,41.9)	40(37.9,42)	39.6(37.5,41.8)	0.009
TP (mg/DL)	67.7(64.4,71.6)	68(64.8,71.9)	67.5(64,71.2)	0.002
PT,s	13.1(12.7,13.7)	13.2(12.7,13.6)	13.1(12.7,13.7)	0.953
D-Dimer, mg/L	0.24(0.17,0.37)	0.23(0.17,0.34)	0.25(0.18,0.39)	<0.001
NT-proBNP, pg/ml	275.2(82,783)	242.5(74.4,706.6)	326(93.3,920.5)	<0.001
Hs-CRP, mg/L	0.84(0.35,2.18)	0.67(0.28,1.57)	1.04(0.44,3.16)	<0.001
MLR	0.20(0.16,0.26)	0.16(0.14,0.18)	0.26(0.23,0.31)	<0.001
MLPR	1.0(0.74,1.40)	0.76(0.61,0.93)	1.35(1.07,1.77)	<0.001
Surgery type, n (%)				
Valvular	1500 (62.8%)	763 (65.9%)	737 (60.0%)	0.003
CABG	706 (29.6%)	257 (22.2%)	449 (36.5%)	<0.001
Congenital	341 (14.3%)	214 (18.5%)	127 (10.3%)	<0.001
Aortic	185 (7.8%)	86 (7.4%)	99 (8.1%)	0.566
Perioperative Variables				
Surgery time(min)	240(198,288)	234(193,278)	246(202,295)	<0.001
CPB time (min)	115(85,147)	111(83,146)	117(88,148)	0.009
Aorta clamp time (min)	81(58,109)	78(55,1047	84(60,111)	0.002
rectal temp	32(31,32.9)	32(3,32.9)	32(31,33)	0.197
Postopetive variables				
Platelets, 10^9/L	210(161,265)	212(166,263)	208(159,266)	0.374
Lymphocyte,10^9/L	0.98(0.70,1.28)	1.07(0.82,1.38)	0.89(0.63,1.16)	<0.001
Monocyte,10^9/L	0.15(0.08,0.26)	0.14(0.08,0.25)	0.17(0.09,0.29)	<0.001
MLR	0.15(0.09,0.25)	0.12(0.07,0.21)	0.19(0.11,0.31)	<0.001
MLPR	0.73(0.40,1.29)	0.59(0.31,1.01)	0.89(0.51,1.56)	<0.001
Urine Volume(48h)	2380(1905,2880)	2380(1870,2850)	2360(1900,2900)	0.851
End point				
AKI	615 (25.8%)	254 (21.9%)	361 (29.4%)	<0.001
length of ICU stays	2.0(1.0-4.0)	2.0(1.0-3.0)	2.0(1.0-4.0)	0.049
length of hospital stays	7(6.0-8.0)	7.0(6.0-8.0)	7.0(6.0-9.0)	<0.001
Death	3 (0.1%)	1 (0.1%)	2 (0.2%)	1
Renal Replace Treatment	11(0.5%)	1(0.1%)	10(0.8%)	0.009

Table 2:Multivariable models for AKI.

	P	OR	Lower Limit	Upper Limit
MLPR(1)	0.004	1.501	1.135	1.987
age	0.041	1.012	1.000	1.024
gender(male)	<0.001	0.518	0.389	0.691
BMI	<0.001	0.927	0.896	0.960
LVEF(30-50%)	<0.001	0.122	0.045	0.330
LVEDD	0.003	1.020	1.006	1.033
NYHA(>50%)	0.002	4.049	1.637	10.016
β-blocker(1)	<0.001	2.510	1.973	3.192
Statin(1)	0.021	0.569	0.353	0.919
Hyperlipidemia(1)	0.049	0.772	0.597	0.998
Previous valvular disease(1)	0.012	1.775	1.136	2.773
Previous congenital disease(1)	0.013	0.491	0.280	0.862
Peripherial vascular disease(1)	<0.001	2.180	1.635	2.905
SCr	<0.001	1.016	1.008	1.024
BUN	0.001	1.109	1.041	1.182
Hs-CRP	<0.001	1.098	1.058	1.139
CPB time	0.038	1.004	1.000	1.008

Figure legends

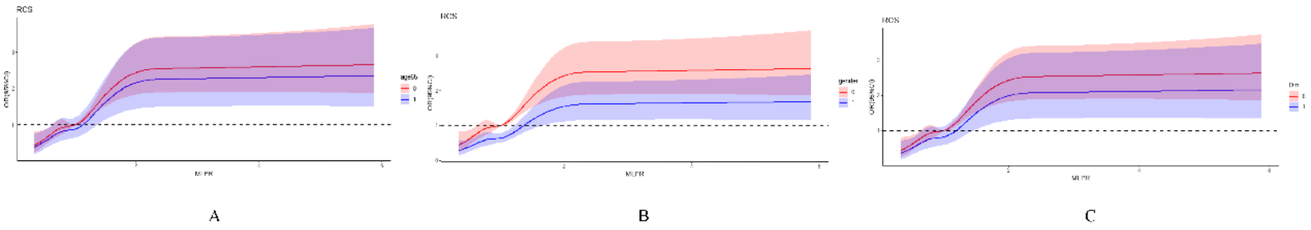

Figure 1: Subgroup analysis for multivariable-adjusted odds ratios of MLPR for AKI based on restricted cubic splines.
(A) Age, red and blue lines represent references for age<65 and age≥65 odds ratios, and red and blue areas represent 95% confidence intervals.
(B)Gender, red and blue lines represent references for female(gender=0) and male (gender=1) odds ratios and red and blue areas represent 95% confidence intervals.
(C) diabetes, red and blue lines represent references for no history of diabetes (Dm=0) and history of diabetes (Dm=1) odds ratios, and red and blue areas represent 95% confidence intervals.
The model was adjusted for age, sex, and previous history of diabetes.

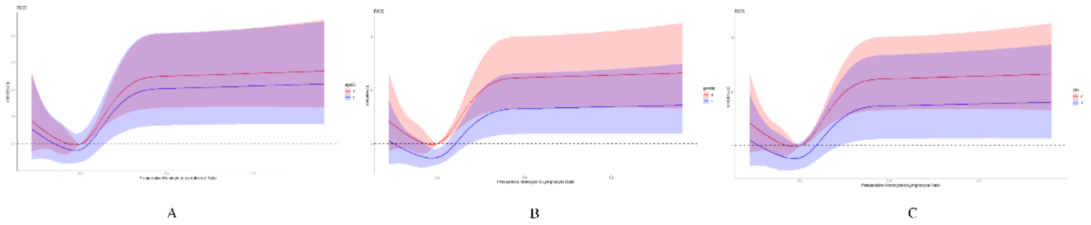

Figure 2: Subgroup analysis for multivariable-adjusted odds ratios of MLR for AKI based on restricted cubic splines.
(A) Age, red and blue lines represent references for age<65 and age≥65 odds ratios, and red and blue areas represent 95% confidence intervals.
(B)Gender, red and blue lines represent references for female(gender=0) and male (gender=1) odds ratios and red and blue areas represent 95% confidence intervals.
(C) diabetes, red and blue lines represent references for no history of diabetes (Dm=0) and history of diabetes (Dm=1) odds ratios, and red and blue areas represent 95% confidence intervals.
The model was adjusted for age, sex, and previous history of diabetes.
